# Clinical characteristics of patients with a family history of psoriasis: an observational epidemiological study in Chinese Han population

**DOI:** 10.3389/fmed.2024.1455953

**Published:** 2024-08-16

**Authors:** Lu Cao, Lingyi Lu, Yingzhe Yu, Huiying Zhou, Bingjiang Lin

**Affiliations:** ^1^Department of Dermatology, The First Affiliated Hospital of Ningbo University, Ningbo, Zhejiang, China; ^2^National Clinical Research Center for Dermatologic and Immunologic Diseases, Beijing, China; ^3^School of Medicine, Ningbo University, Ningbo, Zhejiang, China

**Keywords:** psoriasis, family history, biologics, comorbidities, characteristics

## Abstract

**Introduction:**

Psoriasis, a chronic inflammatory skin disease, is believed to be influenced by both genetic and environmental factors. Despite this understanding, the clinical epidemiological status of psoriasis patients with a family history of the disease remains uncertain.

**Methods:**

In this study, we participated in a multicenter observational epidemiological study involved over 1,000 hospitals and enrolled a total of 5,927 psoriasis patients. These patients were categorized into two groups based on the presence or absence of a family history of psoriasis: family history cases (896) and sporadic cases (5,031). The clinical manifestations of these two groups were analyzed through clinical classification, comorbidities, treatment response, and other relevant factors.

**Results:**

The findings of our study indicate that individuals with a family history of psoriasis predisposition exhibit a notably elevated prevalence of psoriatic arthritis compared to those with sporadic occurrences. Moreover, patients with a family history of psoriasis display a more rapid and efficacious response to secukinumab. Additionally, individuals with moderate to severe psoriasis are at a heightened risk of developing cardiovascular and liver diseases in comparison to those with mild psoriasis, with no discernible impact of familial history on the likelihood of comorbidities.

**Discussion:**

Our study identified the clinical characteristics of individuals with a familial predisposition to psoriasis, offering novel insights into the management and therapeutic approaches for patients with this condition.

## Introduction

Psoriasis is a common chronic inflammatory skin disease characterized by erythematous patches covered with silvery scales. The prevalence of psoriasis ranges from approximately 0.51 to 11.43% (1), the 2019 Global Burden of Disease Study shows that over 4620000 incident cases of psoriasis worldwide (2). Extensive research has demonstrated that psoriasis is a systemic disorder, impacting not only the skin but also various organs such as joints, liver, and kidneys et al. Psoriasis has been shown to increase the prevalence of diseases of inflammatory origin. Increased pro-inflammatory cytokines can be transported from the blood to other systems to produce inflammation and subclinical inflammation, thus therapy for psoriasis could potentially be performed to decrease the risk of comorbidities (3). Additionally, individuals with psoriasis often experience comorbidities, including cardiovascular disease, metabolic syndrome, inflammatory bowel disease, depression, hyperlipidemia and so on (4), Buja et al. have shown that at the onset of psoriasis, patients are more likely to be diagnosed with 2–4 comorbidities or over 5 comorbidities (5). Hence, it is imperative for clinicians to adopt a comprehensive approach in the management of psoriasis patients, considering the potential involvement of other physiological systems and comorbidities, rather than solely concentrating on cutaneous manifestations.

Currently, the etiology of psoriasis remains incompletely understood, with the prevailing perspective suggesting that it is an immune-mediated inflammatory disorder influenced by genetic and environmental factors. The initial identification of the HLA-Cw6 gene, encoding MHC class I receptors involved in antigen presentation to T cells, marked a significant milestone in understanding psoriasis susceptibility (6). Subsequent research has uncovered over 70 additional genetic loci associated with psoriasis (7), offering promising avenues for enhancing clinical diagnosis and personalized drug therapies. The treatment approach for patients with psoriasis may vary depending on the disease severity, with options including local or systemic therapies. A variety of biologic agents have been approved for the treatment of psoriasis in China, including TNF-α inhibitors, IL-23 inhibitors, IL-17A inhibitors, IL12/23 inhibitors, etc. The widespread use of biologics in clinical settings has led to notable enhancements in the quality of life for individuals affected by psoriasis (8).

This study seeks to elucidate the influence of familial history on the epidemiological profile of psoriasis patients within the Chinese demographic, investigate potential disparities in clinical presentations and therapeutic outcomes between psoriasis patients with familial predisposition and those without, and offer novel insights for the clinical assessment and management of individuals with psoriasis.

## Materials and methods

### Questionnaires and patients

A multicenter observational epidemiological study was conducted on psoriasis patients in China, initiated by the National Clinical Research Center for Dermatologic and Immunologic Diseases and involving over 1,000 hospitals. The project has been launched since 2020, and in two years, a total of more than 90,000 patients have been enrolled. As project participants, we applied for part of the data to conduct relevant research. Clinical information was systematically collected using a standardized questionnaire and updated chronologically during each follow-up visit. The questionnaire was structured into baseline and follow-up sections, encompassing fundamental and clinical data, including measurements such as height, weight, age, psoriasis classification, body surface area (BSA) score, psoriasis area and severity index (PASI) score, dermatology life quality index (DLQI) score, comorbidities, previous treatment regimens, and so on. The questionnaire is completed by clinicians in attendance and then uploaded to the system for archival purposes. The comorbidities under investigation encompass cardiovascular disease, diabetes, respiratory disease, liver disease, gastrointestinal disease, rheumato-immune disease, neuropsychiatric disease, tumors, and other skin diseases. A cohort of 7,037 patients with psoriasis was requested for the study, with each patient being diagnosed by two or more dermatologists holding senior professional titles.

Cases with no follow-up records due to time or various reasons and incomplete information related to the study, such as family history, comorbidity and other blank options were excluded, a total of 5,927 individuals were included in the follow analysis. The study population was stratified into two distinct cohorts based on whether there was presence of a familial history of psoriasis: Group 1 comprised individuals with a documented family history of the condition, while Group 2 consisted of sporadic cases, with sample sizes of 896 and 5,031, respectively. Severity of psoriasis was assessed according to the Chinese guidelines for diagnosis and treatment of psoriasis 2018, categorizing patients with DLQI < 6 or PASI < 3 or BSA < 3% as mild, and those not meeting these criteria as moderate to severe. According to the severity of psoriasis, we divided the patients into mild group, named Group 3, and moderate to severe group, named Group 4, with 857 and 5,070 patients, respectively.

Group 1 consisted of 83 patients treated with biologics, while Group 2 included 422 patients, the specific types of biologics and their usage numbers were detailed in Supplementary Table 1. These patients had not previously received biologic therapy for psoriasis, instead, they had received conventional treatments such as topical medications, oral immunosuppressants, or narrowband ultraviolet B (NB-UVB) phototherapy, which had poor response to the control of psoriatic skin lesions. During the analysis of the efficacy of biological agents, patients who received treatment with secukinumab and had follow-up records after 1 month of treatment were selected as the study subjects, based on a comprehensive consideration of follow-up time, the number of patients using various biological agents, and the frequency of biological agent injections, other samples were excluded. Among them, 48 patients in Group 1 and 152 patients in Group 2 received secukinumab injections and had complete follow-up data.

### Statistical analysis

The data analysis was performed using SPSS 20.0 software (SPSS Inc., Chicago, IL, USA). Qualitative and quantitative analyses were conducted on the clinical data. The quantitative data such as age, BMI, BSA, PASI, DLQI, were expressed as mean ± standard deviation, or median (interquartile range) according to whether they fit the normal distribution, while frequency distribution was used in qualitative analysis, like clinical subtypes, sex, and severity. For quantitative data conforming to a normal distribution, t-test was used for analysis. Otherwise rank sum test and chi-square test were used for categorical variables. Rank sum test was used to compare the changes in BSA and PASI scores before and after secukinumab treatment to reflect the effectiveness of biologics. Statistical significance was determined by *P* < 0.05.

## Result

### Clinical manifestations of psoriasis patients with or without family history

The 5927 samples examined in this study were stratified into two cohorts based on the presence or absence of a familial history of psoriasis. Group 1 consisted of 896 samples with a family history, while Group 2 comprised 5031 samples without a familial history. Detailed clinical characteristics of both groups, including age, BMI, BSA, and PASI, are presented in Table 1. During the analysis, it was observed that there were numerous deficiencies in the assessment of DLQI values. Therefore, statistical analysis of this parameter was not conducted.

**TABLE 1:** Clinical characteristics of both groups.

	Group 1 (*n* = 896)	Group 2 (*n* = 5031)
Age (years)	39.24 ± 14.62	42.55 ± 16.01
Sex (male/female)	589/307	3268/1763
BMI (Kg/m^2^)	24.77 ± 5.36	24.33 ± 4.91
BSA (%)	12 (5–30)	11 (5–30)
PASI	9.9 (4.4–17.6)	9 (3.6–16.2)
Severity (mild/moderate to severe)	128/768	729/4302

Chi-square test was performed to analyze the severity and classification of disease in Group 1 and Group 2. The results, presented in Tables 1, 2, indicated no significant difference in disease severity distribution between the two groups. However, a statistically significant difference was observed in disease classification (*p* = 0.039). Patients with a family history of psoriasis were more inclined to exhibit psoriatic arthritis, whereas sporadic patients were more likely to present with pustular psoriasis. The distribution of erythrodermic psoriasis and vulgaris psoriasis was found to be similar in both groups.

**TABLE 2:** Classification of disease of both groups.

	Vulgaris psoriasis	Pustular psoriasis	Erythrodermic psoriasis	Psoriatic arthritis
Group1	859 (95.9%)	11 (1.2%)	14 (1.6%)	12 (1.3%)
Group2	4786 (95.1%)	125 (2.5%)	82 (1.6%)	38 (0.8%)

### Efficacy of biologics

The comparison of BSA and PASI values before and after treatment (Baseline and Week 4 data) revealed that the decline in BSA and PASI in Group 1 was significantly greater than that in Group 2, as indicated by the rank sum test for the difference values. Specifically, the mean declines in BSA and PASI in Group 1 were 22.29 and 11.21, respectively, compared to 14.10 and 7.67 in Group 2 (*P*_bsa_ = 0.029, *P*_pasi_ = 0.044). These results suggest that the efficacy of secukinumab treatment for psoriasis patients with a family history is more pronounced.

### Comorbidities with psoriasis

**FIGURE 1:**
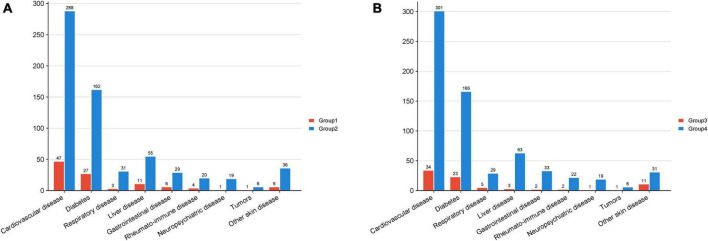
The amount of comorbidity cases in each group. The horizontal axis denotes the various comorbidities, while the vertical axis indicates the quantity of patients. **(A)** Group 1 pertains to patients with a familial history of psoriasis, Group 2 pertains to patients with sporadic psoriasis. **(B)** Group 3 pertains to patients with mild psoriasis, and Group 4 pertains to patients with moderate to severe psoriasis.

The study examined the presence of comorbidities among patients diagnosed with psoriasis in Group1 and Group 2, the specific types and frequencies of comorbidities are detailed in Table 3 and Figure 1, with cardiovascular diseases being the most prevalent comorbidity in both Group 1 (5.2%) and Group 2 (5.7%). A Chi-square test was conducted to compare the distribution of comorbidities between the two groups, indicating no statistically significant difference. Thus, it can be concluded that family history did not contribute to an increased predisposition to comorbidity prevalence.

**TABLE 3:** The specific types and amount of comorbidities.

	Form of disease			Severity		
	Group 1 (*n* = 896) (%)	Group 2 (*n* = 5031) (%)	χ2 test (*P*-value)	Group 3 (*n* = 857) (%)	Group 4 (*n* = 5070) (%)	χ2 test (*P*-value)
Cardiovascular disease	47 (5.2)	288 (5.7)	0.637	34 (4.0)	301 (5.9)	0.020*
Diabetes	27 (3.0)	162 (3.2)	0.837	23 (2.7)	166 (3.3)	0.402
Respiratory disease	3 (0.3)	31 (0.6)	0.469	5 (0.6)	29 (0.6)	1.000
Liver disease	11 (1.2)	55 (1.1)	0.729	3 (0.4)	63 (1.2)	0.020*
Gastrointestinal disease	6 (0.7)	29 (0.6)	0.642	2 (0.2)	33 (0.7)	0.223
Rheumato-immune disease	4 (0.4)	20 (0.4)	0.776	2 (0.2)	22 (0.4)	0.565
Neuropsychiatric disease	1 (0.1)	19 (0.4)	0.345	1 (0.1)	19 (0.4)	0.344
Tumors	1 (0.1)	6 (0.1)	1.000	1 (0.1)	6 (0.1)	1.000

*p < 0.05.

The samples were reclassified according to the severity of psoriasis, resulting in 857 individuals classified as mild patients and 5,070 individuals classified as moderate to severe patients, designated as Group3 and Group4, respectively. A Chi-square test was performed to analyze the prevalence of comorbidities among the patients, revealing that the proportion of moderate to severe patients with cardiovascular disease and liver disease was significantly higher compared to mild patients (*P* = 0.020 and *P* = 0.020, respectively). This suggests that moderate and severe psoriasis patients are more susceptible to cardiovascular disease and liver disease than those with mild psoriasis, with no similar predisposition observed for other conditions.

## Discussion

Our study found a significant correlation between the presence of a family genetic history and the type of psoriasis. Specifically, the proportion of patients with a family history of psoriasis exhibiting psoriatic arthritis was notably higher than that of sporadic cases, while the prevalence of pustular psoriasis was higher among sporadic cases compared to those with a family history. Psoriasis vulgaris was the most common type in both groups. Studies in the Japanese population have also found that psoriatic arthritis was significantly more common in patients with a family history (9), psoriatic arthritis usually involves the peripheral joints, axial skeleton, and periarticular (10). Several previous studies have also investigated the clinical manifestations of psoriasis in patients with a family history, research from Canada and Turkey had shown that people with a family history of psoriasis or psoriatic arthritis were more likely to be women, had an earlier age of onset, and had more frequent nail involvement, joint deformities, and presence of enthesitis. Furthermore, patients with a family history of psoriatic arthritis had lower risk for plaque psoriasis (11). Additionally, several literatures had also documented a higher proportion of nail involvement in patients with a positive family history (12, 13). Therefore, it is important to focus on whether there are joint and nail involvement in the clinical diagnosis and treatment of patients with a family history of psoriasis. Duffin et al. demonstrated that family history of psoriasis was a predisposing factor for guttate psoriasis, however we did not disaggregate statistics for psoriasis vulgaris, it is not clear whether the same association exists in the Chinese population (14).

Given the chronic and recurrent nature of psoriasis, it remains a challenging condition for both healthcare providers and patients (15). The use of biologic has enabled the effective treatment of lesions in individuals with moderate to severe psoriasis, with several biologics receiving approval from the FDA, including IL-23 inhibitors, IL-17A inhibitors, and IL-12/23 inhibitors. Among these, secukinumab, a fully human IL-17A monoclonal antibody, has been sanctioned for the management of plaque psoriasis, psoriatic arthritis, ankylosing spondylitis, among other conditions. Our research findings indicate that patients with a familial predisposition to psoriasis exhibit a more robust response to secukinumab.

In light of this conclusion, we have posited two hypotheses. The first hypothesis suggests that individuals with psoriasis who have a familial predisposition may exhibit shared genetic characteristics that facilitate a more expedited and effective response to secukinumab, thereby enabling the categorization of the disease based on molecularly informed taxonomy (16), a methodology frequently employed in the study of respiratory ailments (17). Recent research has indicated that the efficacy of secukinumab treatment is not influenced by common genetic variants, HLA alleles, or other genetic factors (18). As a multifaceted condition, the genetic underpinnings of psoriasis extend beyond the polygenic risk scores of identified susceptibility genes (19). The reliability of these scores for non-European populations remains uncertain, as does the utility of group identification in this context. Furthermore, the effectiveness of secukinumab appears consistent across diverse genetic backgrounds and does not equate to a subset of the population responding more quickly or efficiently. Additionally, the enhanced or expedited response to secukinumab may be linked to psoriatic subtypes. Our study revealed that a greater percentage of individuals with a familial predisposition to psoriasis were categorized as having psoriatic arthritis in comparison to those with sporadic cases. Secuchiumab has been shown to inhibit the progression of psoriatic arthritis at the radiographic level, and is recommended as a treatment for psoriatic arthritis in multiple international and domestic guidelines. Moreover, IL-17A inhibitors demonstrated a more favorable efficacy in treating psoriatic arthritis than pustular psoriasis (20). Therefore, patients with a family history of psoriasis show a stronger response to secuchiumab, however, whether this reaction is specific to secuchiumab or to all biologics needs further study. In 2020, a dichotomous definition was developed by the International Psoriasis Council, that is, based on BSA size, whether special sites are involved and whether local treatment is effective to define psoriasis severity (21). Since early selection of appropriate treatments can provide both immediate and long-term benefits (22), based on the clinical characteristics of psoriasis patients with a family history and the effective response to secuchiumab were found in this study, we suggest that patients with a family history of psoriasis may try to initiate biologics as early as possible.

Our research findings indicate that individuals with moderate to severe psoriasis exhibit notably elevated incidences of cardiovascular and liver diseases compared to those with mild psoriasis. Specifically, the prevalence of cardiovascular disease is most pronounced in cases of hypertension and coronary heart disease. Existing literature has established a link between chronic systemic inflammation and the pathogenesis of cardiovascular disease (23–25). Individuals diagnosed with psoriasis demonstrated an odds ratio of 2.18 for developing atherosclerosis and a 6% elevation in the Framingham risk score (26). The adipose tissue of psoriasis patients harbors a significant quantity of immune cells linked to cardiometabolic function, including T cells, B cells, dendritic cells, mast cells, and adipose tissue macrophages. These cells play a role in the pathogenesis of obesity and insulin resistance, producing pro-inflammatory cytokines and adipose tissue dysfunction, which are implicated in the advancement of atherosclerotic vascular disease (27). In addition, there are many links between immune cell dysfunction in psoriasis patients and the progression of cardiovascular disease (28).

Previous research has shown a positive correlation between the severity of psoriasis and the occurrence of cardiovascular events. The analysis of computed tomography scans of individuals with psoriasis, showed a notable increase in both the rate and severity of coronary artery calcification among these patients, with a direct correlation to the severity of their psoriasis (29). Additionally, research has demonstrated that patients with psoriasis who do not have cardiovascular disease exhibit a diminished Coronary flow reserve (CFR), suggesting the presence of coronary microvascular dysfunction. This finding is associated with the severity and advancement of the disease (30). The TH1/TH17 axis is widely recognized for its significant involvement in the pathogenesis of psoriasis, with the progression of atherosclerosis being closely linked to the secretion of IL-17A by TH17 cells. Consequently, the susceptibility of individuals with psoriasis to cardiovascular events may be intricately tied to the underlying pathophysiology of the disease (31). Furthermore, Su et al. demonstrated that psoriasis and atherosclerosis exhibit a shared differential expression of up to 16 genes, offering a novel insight into the investigation of the mutual pathogenesis of these two conditions (32).

As for the other comorbidities associated with severity, liver disease, non-alcoholic fatty liver disease (NAFLD) is the predominant chronic liver condition, encompassing non-alcoholic fatty liver, non-alcoholic steatohepatitis, cirrhosis, and other related conditions. It is prevalent in approximately 30% of adults residing in Western nations (33). Prior research has established a correlation between non-alcoholic fatty liver disease and psoriasis (4, 34), with psoriasis patients exhibiting a 1.5–3 times higher incidence of NAFLD compared to other populations (35). This association is believed to be linked to metabolic syndrome and systemic inflammation (36), and psoriasis patients with NAFLD tend to experience higher disease activity and PASI scores (37).

Interestingly, studies have also shown an inextricable link between cardiovascular disease and liver disease. Studies have indicated that NAFLD is a significant risk factor for the development of atherosclerosis. Originally believed to be a manifestation of metabolic syndrome in the liver, NAFLD is now understood to contribute to the progression of atherosclerosis through various mechanisms, including the secretion of pro-inflammatory cytokines such as TNF-α and IL-6 (38). For instance, TNF-α facilitates the proliferation of keratinocytes in psoriasis, enhances the uptake of low-density lipoprotein in endothelial cells leading to the deposition of blood vessel walls in cardiovascular disease, and concurrently disrupts insulin metabolism, a factor closely associated with the development of non-alcoholic fatty liver disease (39–41).

As research progresses, mounting evidence indicates that NAFLD is an autonomous risk factor for cardiovascular disease (CVD). NAFLD is implicated in oxidative stress, chronic inflammation, dyslipidemia, and other physiological processes (42–44). Hence, clinicians should consider the potential presence of comorbidities in psoriasis patients, particularly those with moderate to severe psoriasis, and prioritize enhanced screening and prevention measures for cardiovascular and liver diseases.

## Conclusion

Our research delineates the clinical features of individuals with a familial predisposition to psoriasis and demonstrates that those with a familial history exhibit enhanced responsiveness to secuchiumab treatment. Additionally, individuals with moderate to severe psoriasis are more needed to undergo screening for cardiovascular and hepatic risks. These findings offer novel insights for the diagnostic and therapeutic management of psoriasis.

## Data Availability

The original contributions presented in the study are included in the article/Supplementary material, further inquiries can be directed to the corresponding author.
