# Supplementary material for: Clinical characteristics of patients with a family history of psoriasis: an observational epidemiological study in Chinese Han population
Source: Front Med (Lausanne). 2024 Aug 16;11:1455953. doi: 10.3389/fmed.2024.1455953 (PMC11362089; doi:10.3389/fmed.2024.1455953)
Supplement: Supplementary file 1 [file Data_Sheet_1.pdf]

Supplementary table1. The number of users of various biological agents

	anti-TNF-α			anti-IL-17A		anti-IL-12/23	anti-IL-23	Others
	Adalimumab	Etanercept	Infiximab	Secukinumab	Ixekizumab	Ustekinumab	Guselkumab	
Group 1 n = 83	16	1	2	57	5	0	0	2
Group 2 n = 422	78	13	15	184	42	47	18	25
